# A Digitally Enabled Combined Lifestyle Intervention for Weight Loss: Pilot Study in a Dutch General Population Cohort

**DOI:** 10.2196/38891

**Published:** 2024-02-08

**Authors:** Rahul Gannamani, José Castela Forte, Pytrik Folkertsma, Sven Hermans, Sridhar Kumaraswamy, Sipko van Dam, Niels Chavannes, Hendrikus van Os, Hanno Pijl, Bruce H R Wolffenbuttel

**Affiliations:** 1 Ancora Health BV Groningen Netherlands; 2 Department of Neurology University Medical Centre Groningen University of Groningen Groningen Netherlands; 3 Department of Clinical Pharmacy and Pharmacology University Medical Centre Groningen University of Groningen Groningen Netherlands; 4 Department of Endocrinology University Medical Centre Groningen University of Groningen Groningen Netherlands; 5 Department of Public Health and Primary Care Leiden University Medical Centre Leiden University Leiden Netherlands; 6 National eHealth Living Lab Leiden Netherlands; 7 Department of Endocrinology Leiden University Medical Center Leiden University Leiden Netherlands

**Keywords:** lifestyle intervention, prevention, obesity, overweight, weight loss, digital health, intervention, weight, pilot, digital, data, Fogg Behavior Model

## Abstract

**Background:**

Overweight and obesity rates among the general population of the Netherlands keep increasing. Combined lifestyle interventions (CLIs) focused on physical activity, nutrition, sleep, and stress management can be effective in reducing weight and improving health behaviors. Currently available CLIs for weight loss (CLI-WLs) in the Netherlands consist of face-to-face and community-based sessions, which face scalability challenges. A digitally enabled CLI-WL with digital and human components may provide a solution for this challenge; however, the feasibility of such an intervention has not yet been assessed in the Netherlands.

**Objective:**

The aim of this study was two-fold: (1) to determine how weight and other secondary cardiometabolic outcomes (lipids and blood pressure) change over time in a Dutch population with overweight or obesity and cardiometabolic risk participating in a pilot digitally enabled CLI-WL and (2) to collect feedback from participants to guide the further development of future iterations of the intervention.

**Methods:**

Participants followed a 16-week digitally enabled lifestyle coaching program rooted in the Fogg Behavior Model, focused on nutrition, physical activity, and other health behaviors, from January 2020 to December 2021. Participants could access the digital app to register and track health behaviors, weight, and anthropometrics data at any time. We retrospectively analyzed changes in weight, blood pressure, and lipids for remeasured users. Surveys and semistructured interviews were conducted to assess critical positive and improvement points reported by participants and health care professionals.

**Results:**

Of the 420 participants evaluated at baseline, 53 participated in the pilot. Of these, 37 (70%) were classified as overweight and 16 (30%) had obesity. Mean weight loss of 4.2% occurred at a median of 10 months postintervention. The subpopulation with obesity (n=16) showed a 5.6% weight loss on average. Total cholesterol decreased by 10.2% and low-density lipoprotein cholesterol decreased by 12.9% on average. Systolic and diastolic blood pressure decreased by 3.5% and 7.5%, respectively. Participants identified the possibility of setting clear action plans to work toward and the multiple weekly touch points with coaches as two of the most positive and distinctive components of the digitally enabled intervention. Surveys and interviews demonstrated that the digital implementation of a CLI-WL is feasible and well-received by both participants and health care professionals.

**Conclusions:**

Albeit preliminary, these findings suggest that a behavioral lifestyle program with a digital component can achieve greater weight loss than reported for currently available offline CLI-WLs. Thus, a digitally enabled CLI-WL is feasible and may be a scalable alternative to offline CLI-WL programs. Evidence from future studies in a Dutch population may help elucidate the mechanisms behind the effectiveness of a digitally enabled CLI-WL.

## Introduction

### Background

The morbidity and mortality burden associated with obesity, diabetes, and cardiovascular disease (CVD) continues to increase globally [1]. The prevalence of diabetes and CVD has nearly doubled since 1990 to over 536 and 520 million cases worldwide, respectively [1,2]. Obesity, as a major risk factor for CVD and diabetes, is also associated with a decrease in life expectancy between 5 and 20 years, depending on the severity of the condition and comorbid disorders [3]. In addition, overweight-related diseases are expected to give rise to treatment costs equivalent to 8.4% of health spending in Organization for Economic Cooperation and Development countries [4].

A substantial portion of the risk of diabetes, CVD, and obesity is attributable to modifiable lifestyle factors such as an unhealthy diet, lack of physical activity, and smoking, which subsequently lead to metabolic imbalances and overweight or obesity [1-3]. Overweight and obesity are often the direct result of a disturbed energy balance, generated through a combination of the above-mentioned factors as well as excessive dietary energy consumption [5,6]. Despite being common knowledge, the principles of living a healthy lifestyle are insufficiently followed by the general population. Indeed, recent data show that over 50% of the Dutch population do not meet daily activity guidelines and that many elements of an individual’s environment are often obesogenic [7,8]. In addition, other health behavioral aspects such as deficient sleep and poor stress management also increase the risk of developing excess weight [9-11]. It is therefore unsurprising that overweight and obesity rates among the general population of the Netherlands keep increasing: in 2021, almost 50% of Dutch adults were living with overweight and 13% were living with obesity [12].

Therefore, successful approaches to preventing and treating overweight/obesity, CVD, and associated risk factors ought to tackle at least the four essential lifestyle components of nutrition, activity, sleep, and stress management, along with smoking and alcohol consumption when possible [13-15]. Preferably, this is done in a personalized way, since different individuals will be exposed to different triggers and face different challenges that lead to excess weight or failure to lose weight [16,17]. Combined lifestyle interventions (CLIs), especially those targeting weight loss (CLI-WLs), provide a potential solution for people who are overweight or obese to initiate and maintain healthier lifestyle behaviors [18]. Since 2019, some CLI-WL programs have been covered by basic insurance in the Netherlands for individuals meeting prespecified criteria such as obesity in case of a BMI≥30 or overweight and increased risk of comorbidities, including diabetes and CVD [19]. Dutch CLI-WL programs are 2-year programs delivered at varying intensities throughout this period, which consist of guidance mostly on physical activity and nutrition [18]. These programs consist of both individual and group sessions to educate participants, allowing them to share experiences and provide support [18]. The program is typically carried out completely offline by lifestyle coaches, physiotherapists, and dietitians accredited to deliver CLI-WL to patients referred by general practitioners [18]. Internationally, interventions similar to the Dutch CLI-WL have been shown to be effective in terms of weight reduction and health improvements, even when compared with standard care or pharmacological treatment [20]. However, reports on the effectiveness of Dutch CLI-WL programs since their inclusion in the basic insurance package have been inconsistent, with the effects of the interventions either falling short of expectations or not structurally translating to sustained weight loss in the medium to long term [21-23]. Previous studies have shown that the biggest barrier experienced by participants is the challenge of implementing and following lifestyle changes in a sustainable way [23,24].

A growing number of digital programs that can support individuals and providers in addressing health risks and conditions are being developed and made available to the public [25]. These so-called digital therapeutic solutions stem from an emerging field at the cross-section between health care and technology. Similar to pharmacological therapies or hardware medical devices, these therapeutics are evidence-based software products for the prevention, management, and treatment of health conditions. Several studies suggest that interventions including digital elements such as remote data monitoring and the possibility to digitally communicate with providers are associated with higher engagement from participants and subsequently with better health outcomes [26,27]. The incorporation of these digital elements—which are considered critical components of successful behavior change programs—can therefore help overcome the cited barriers to engagement in face-to-face programs such as accessibility, transportation, and scheduling [24,25,28]. In fact, digital therapeutic solutions, ranging from digital weight loss programs based on intensive dietary coaching to tracking and gamification apps, have been shown to induce weight loss to varying degrees, surpassing what offline interventions achieve [29,30]. Therefore, deploying a digitally enabled CLI-WL could broaden access to prevention and care, while delivering superior or at least comparable outcomes to those reported for currently available, completely offline CLI-WL programs in the Netherlands. Due to the novelty of digitally enabled CLI-WL programs in the Netherlands, there is a research gap that we seek to bridge with this research.

### Objectives

The objective of this pilot study was to provide a real-world contribution to the discussion surrounding the feasibility of digitally enabled CLI-WL programs in the Netherlands. In this study, we assessed the changes in weight and concomitant cardiometabolic risk factors of a cohort of Dutch adults with overweight or obesity and cardiometabolic risk who participated in the pilot of a digitally enabled CLI-WL. In addition, we also conducted surveys and semistructured interviews to assess the critical positive and improvement points reported by participants and health coaches to further guide the development of future iterations of the intervention.

## Methods

### Study Sample

**Figure 1:**
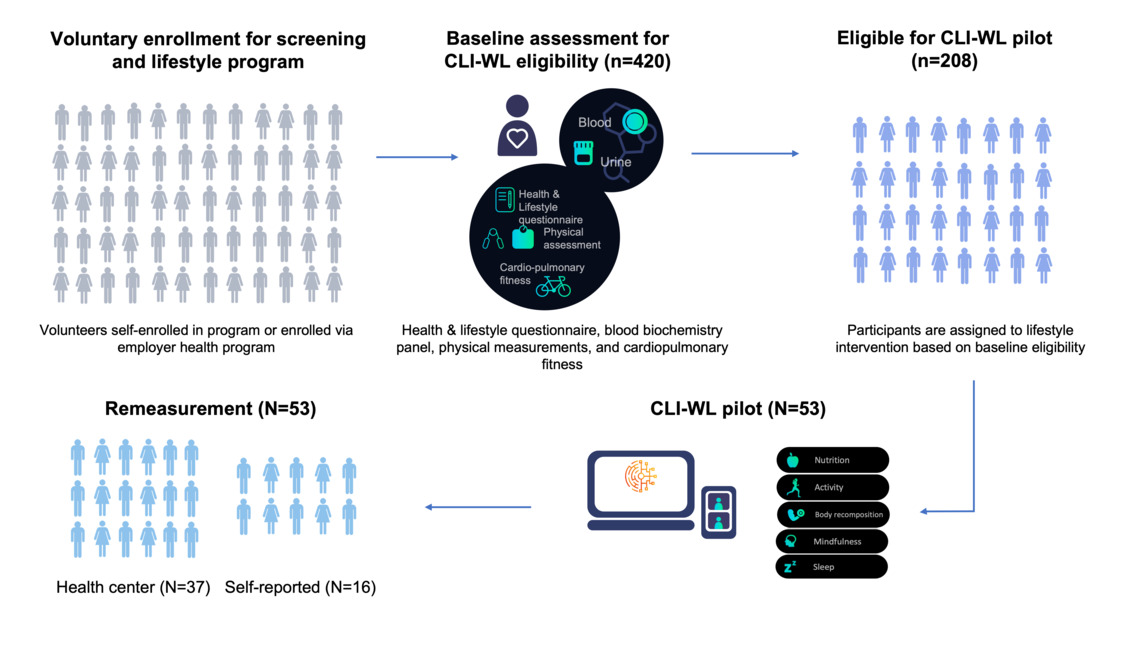
Overview of the study flow, including sample size at each stage. CLI-WL: combined lifestyle intervention targeting weight loss.

As of December 2021, 420 users were enrolled in the Ancora Health Lifestyle program through employer health programs or as a direct-to-consumer option. A study on a subset of this cohort was published previously in *JMIR Cardio* [31]. Body weight, blood pressure, and lipids (total and low-density lipoprotein [LDL] cholesterol) were measured at baseline. Participants who were classified as obese (BMI≥30) or overweight (BMI≥25) with one or more cardiometabolic risk factors were asked to participate in a 16-week digitally enabled CLI consisting of digital and human components. The cardiometabolic risk factors considered were a diastolic blood pressure≥80 mmHg or systolic blood pressure≥130 mmHg, dyslipidemia (LDL cholesterol≥3.0 mmol/L, total cholesterol≥5.1 mmol/L, high-density lipoprotein cholesterol<1 mmol/L, or triglycerides≥1.8 mmol/L), or prediabetes (hemoglobin A1c≥5.7% or fasting glucose≥5.6 mmol/L). An overview of the study flow is given in Figure 1.

### Combined Lifestyle Intervention

After baseline measurements, users were provided access to a web-based digital app where they could register and track health behaviors, weight, and anthropometrics data at any time. This app is a certified Class I medical device that generates evidence-based lifestyle recommendations spanning across nutrition, physical activity, sleep improvement, stress reduction, and tracking behaviors (such as logging weight) that are tailored to the individual’s prevailing risk profile. Other preliminary evidence of the health benefits of lifestyle interventions delivered to a subset of this cohort with the Ancora Health platform for health goals other than weight loss (eg, lipids, blood pressure, and nutrient imbalances) has been published previously [32-34].

The intervention was initiated with a 30-minute intake consultation conducted by video call. The intake consultation provided counseling on health risks, recommendation of targeted lifestyle medicine actions, and a “handshake” to undertake these actions for the following period. During the intervention, coaching was delivered digitally through one-on-one chat-based contact with optional audio/video calls alongside this format. Coaching was delivered by a health care professional with a background in either lifestyle coaching, nutrition, physiotherapy, or psychology, depending on the prevailing behavioral coaching required. This coaching was complemented by weekly progress reports. This approach builds on the Fogg Behavioral Model (FBM) [35]. The FBM posits that behavior change occurs when users are prompted to perform target behaviors that they are sufficiently motivated and sufficiently able to perform, with a trade-off between the level of motivation and level of ability. In this intervention, the FBM was implemented proactively through digital coaching: coaches used motivational interviewing techniques to manage/positively influence participants’ motivation levels and adjusted the difficulty of target behaviors on an ongoing basis in line with the participants’ motivation and/or skill level. Moreover, coaches helped users with tips/tricks and strategies to overcome any barriers encountered.

### Measurements at Intake, During the Program, and After the Program

Upon enrollment to the program, participants underwent a baseline assessment where a comprehensive lifestyle questionnaire, a blood biochemistry panel, and physical measurements were collected using the InBody model 570 for body composition and the InBody BIOBP750 cuff for blood pressure. After the baseline assessment, users could access the digital web app to register and track their health behaviors and modify weight data at any time during the intervention. At follow-up after the intervention, which participants could voluntarily enroll for, the subset of blood biochemistry parameters found to be abnormal at baseline and the lifestyle questionnaire and physical measurements were reassessed. Weight and other cardiometabolic risk factors were remeasured at the health center for participants who were able to return for a remeasurement. Participants not able to return were asked to self-report their weight after receiving instructions from their lifestyle coaches (namely to measure it in the morning, before eating) (Figure 1). For self-reporting participants, other markers were not remeasured. According to the definition used in the criteria for participation in a CLI-WL in the Netherlands, we classified BMI values between 25 and 30 as “overweight” and values greater than or equal to 30 as “obese.” Changes from baseline in weight, blood pressure, and lipid markers were calculated by subtracting the end values from the first reported values, and the percent change was calculated by dividing the observed change by the baseline value.

After the entire cohort completed their intervention, the first author conducted semistructured interviews with 17 participants and 6 of the coaches delivering the intervention, either physically or via audio/video in Dutch or English. Semistructured interviews allow for greater flexibility for both the interviewer and participant than traditional, structured interviewing, while simultaneously providing greater direction in the interview process than completely unstructured interviews. The user research framework reported in this study was based on guidelines published by the Medical Research Council for process evaluations [36] and the Conceptual Framework for Implementation Research [37,38]. The focus of the interviews was acceptability and accessibility, two critical process indicators in the adoption of digital interventions. While there is no consensus definition of acceptability, it can be broadly defined as “people’s affective attitudes toward a new digital health intervention,” “usage intentions or actual usage,” and “satisfaction after having engaged with the intervention” [39]. Since most available acceptability measures in pilot or feasibility studies of digital health interventions lack a theoretically or empirically established cutoff, it has been suggested that 1 to 5 ratings and accompanying free-text responses may provide a sufficiently precise measure of acceptability [40].

The interview guide had questions for the coaches and participants focused on their experience coaching or partaking in the intervention, and on what they thought were the most important positive and improvement points for the quality of the intervention.

### Statistical Analysis

Descriptive statistics were calculated to characterize the population at baseline in terms of demographics and clinical parameters. Paired remeasurement versus baseline changes in weight, blood pressure, and cholesterol in participants who were remeasured after a median of 10 months were assessed with the Student *t* test or Wilcoxon signed-rank test depending whether or not the data were normally distributed. All categorical variables are reported as percentages and continuous variables are reported as mean and SD. The *χ*^2^ test and analysis of variance were used to evaluate differences in categorical and continuous variables, respectively, at the cohort level. We considered *P*<.05 to indicate a statistically significant difference between cohorts and in pre- and postintervention measurements. All data analyses were performed using R software v4.0.3. Themes and coach/participant opinions were registered following an inductive process after the main findings of the interviews conducted by the first author were discussed in a multidisciplinary setting with the coaches, with no preexisting framework or theoretical constructs used to classify data [41,42].

### Ethics Approval

The study was declared exempt from institutional review board approval through a waiver issued by the Medical Ethical Committee of the University Medical Centre Groningen (waiver number METC#2021/488).

## Results

### Baseline Characteristics

Baseline characteristics of the total study sample are shown in Table 1. We found that 208 of the 420 participants (49.5%) were classified as either obese or overweight with one or more cardiometabolic risk factors. Of these, 53 participants enrolled in the pilot study. These individuals were older, had higher lipid levels and blood pressure, as well as higher weight and BMI compared to the rest of the cohort (Table 1).

**Table 1:** Baseline characteristics of the total study sample and the participants who enrolled in the pilot study.

Characteristics			Entire cohort (N=420)		Participants of the pilot study (n=53)		*P* value^a^
**Demographics**							
	Age (years), mean (SD)	44.6 (11.1)		48.2 (9.0)		.007	
	Female sex, n (%)	227 (54.0)		29 (54.7)		.99	
**Anthropometrics, mean (SD)**							
	Weight (kg)	77.5 (14.4)		87.2 (11.2)		<.001	
	BMI (kg/m²)	25.0 (4.6)		29.1 (3.4)		<.001	
**Blood pressure (mmHg), mean (SD)**							
	Systolic	131 (17)		146 (15)		<.001	
	Diastolic	81 (12)		93 (11)		<.001	
**Lipids (** **mmol/L), mean (SD)**							
	Total cholesterol	5.1 (1.1)		6.4 (1.0)		<.001	
	Low-density lipoprotein cholesterol	3.1 (0.9)		4.3 (0.9)		<.001	

^a^Unpaired *t* test between the entire cohort and the pilot group.

### Baseline Values and Changes in Weight and Cardiometabolic Risk Factors

We analyzed weight data for the entire group of 53 participants in the pilot and for the two subsets of participants with overweight and obesity separately (Table 2). In the entire group, the average weight loss achieved was 3.7 kilograms, which equates to an average of 4.2% body weight loss (*P*<.001). In total, 25 individuals (47%) achieved a reduction of at least 5% body weight. In the 37 participants with overweight, the mean weight loss was 2.9 kilograms (3.5% change). For the 16 participants with obesity, weight loss was higher at –5.4 kilograms and –5.6% body weight with a mean baseline weight of 97.3 kilograms (*P*<.001; Table 2).

We also analyzed the changes in cardiometabolic risk factors in participants whose weight was remeasured on location and had abnormal lipid or blood pressure levels at baseline (Table 3). In these participants, total cholesterol, LDL cholesterol, systolic blood pressure, and diastolic blood pressure significantly decreased. In participants with obesity, changes in total and LDL cholesterol as well as in systolic and diastolic blood pressure were not significant, which was attributed to the very small sample size. In participants with overweight, total cholesterol, LDL cholesterol, and diastolic blood pressure were significantly decreased, whereas there was no significant decrease in systolic blood pressure.

None of the participants who were remeasured on location, based on information provided in the medical and lifestyle questionnaire at baseline and follow-up, had initiated blood pressure– or cholesterol-lowering medication during the lifestyle intervention.

**Table 2:** Changes in weight after the combined lifestyle intervention for weight loss.

Group	Preintervention weight (kg), mean (SD)	Postintervention weight (kg), mean (SD)	Absolute (relative) weight change, kg (%)	*P* value
Entire cohort (N=53)	87.2 (11.2)	83.5 (12.0)	–3.7 (–4.2)	<.001
Participants classified as overweight (n=37)	82.8 (8.3)	79.9 (10.4)	–2.9 (–3.5)	<.001
Participants classified as obese (n=16)	97.3 (10.5)	91.9 (11.3)	–5.4 (–5.6)	<.001

**Table 3:** Changes in lipid profile and blood pressure after the combined lifestyle intervention in individuals with abnormal baseline values.

Variable		Preintervention, mean (SD)	Postintervention, mean (SD)	Absolute (relative) change, kg (%)	*P* value
**Total cholesterol (mmol/L)**					
	Entire remeasured group (N=20)	6.38 (1.00)	5.73 (0.92)	–0.65 (–10.2)	.02^a^
	Participants with overweight (n=18)	6.46 (1.01)	5.71 (0.97)	–0.75 (–11.6)	.01^a^
	Participants with obesity (n=2)	5.75 (0.74)	5.88 (0)	0.13 (2.3)	>.99^a^
**LDL^b^ cholesterol (mmol/L)**					
	Entire remeasured group (N=24)	4.34 (0.86)	3.78 (0.88)	–0.56 (–12.9)	.007
	Participants with overweight (n=20)	4.46 (0.86)	3.89 (0.85)	–0.57 (–12.8)	.02
	Participants with obesity (n=4)	3.71 (0.56)	3.18 (0.88)	–0.53 (–14.3)	.17
**Systolic blood pressure (mmHg)**					
	Entire remeasured group (N=22)	146 (15)	141 (16)	–5 (–3.5)	.04^a^
	Participants with overweight (n=18)	148 (16)	143 (16)	–5 (–3.4)	.12^a^
	Participants with obesity (n=4)	139 (6)	131 (14)	–9 (–6.5)	.14
**Diastolic blood pressure (mmHg)**					
	Entire remeasured group (N=26)	93 (11)	86 (12)	–7 (–7.5)	.003^a^
	Participants with overweight (n=19)	94 (11)	86 (12)	–8 (–8.5%)	.003
	Participants with obesity (n=7)	90 (10)	85 (12)	–5 (–5.6%)	.18^a^

^a^Based on the Wilcoxon signed-rank test owing to baseline data not following the normal distribution.

^b^LDL: low-density lipoprotein.

### Quantitative and Qualitative Participant Feedback

The subjective, qualitative feedback collected through surveys (n=37) and semistructured interviews (n=17) from pilot participants is presented in Textbox 1.

The quantitative results of the participant survey that was filled out at the end of the intervention period are presented in Table 4. The highest scoring items were linked to the feeling of involvement in the program (item 1, score 4.5/5), the interaction with the coaches (item 9, 4.6/5), and the knowledge displayed by the coaches (item 10, 4.4/5). Importantly, participants also overwhelmingly expressed the wish to continue working toward their health goals after the intervention (item 4, 4.7/5). The items that scored the lowest were linked to the need for one-on-one coaching and the importance of coaching in achieving the proposed health goals (items 7 and 8, 3.7/5 and 3.8/5, respectively).

The subjective feedback provided during semistructured interviews by the coaches (N=6) is presented in Textbox 2. In general, interviewees valued several aspects as the most positive and differentiating factors of the digitally enabled CLI-WL. These included the possibility of setting clear action plans (goals) for participants to work toward; the promotion of healthy eating, exercise, and other lifestyle habits as opposed to enforcing strict diets; and the speedy, problem solving–oriented interactions across the multiple weekly touch points. Both improvement points were related to the intervention curriculum, with health care professionals suggesting improvements in the food-tracking capabilities of the app as well as an expansion of the coaching resources.

**Summarized feedback collected from pilot participants through surveys and semistructured interviews regarding the acceptability and accessibility of the program.**

Positive points

- Coaches were friendly, engaged, and approachable for guidance
- Proactive check-ins
- Good, personal advice; coaches helped find solutions to overcome barriers
- Program was (positively) challenging
- Effectively motivated for behavioral change
- Provided support in making effective lifestyle changes

Improvement points

- Expand the features in the app (tracking, connectivity, reminders, personalized content)
- More coaching conversations instead of chat messages

**Table 4:** Quantitative results of the participant survey (N=37).

Item	Score^a^
I felt involved in the program right from the start	4.5
I found the stepwise approach to behavioral change in the program easy to follow	4.1
I was motivated to work toward my health goals	4.4
I would like to continue working toward my health goals	4.7
I feel my health and well-being have improved since I participated in the Ancora program	3.9
I found the content of the program relevant and engaging	4.0
Human coaching was important for me to overcome my health challenges	3.7
Human coaching was important for me to achieve my health missions	3.8
I found my coach(es) friendly and empathic	4.6
I found my coach(es) knowledgeable	4.4

^a^ Scoring runs from 1 (“disagree completely”) to 5 (“agree completely”), with 3 being “neutral.”

**Summary of the feedback regarding subjective intervention parameters “acceptability and accessibility” provided by coaches (N=6).**

Positive points

- Possibility to set clear goals for the intervention period based on a holistic assessment of the participant’s health
- Integrative approach that promotes healthy eating and exercise as opposed to strict diets, including specific elements such as strength-training advice, stress management, and good sleep habits
- Multiple touch points weekly between the Health Engagement team to enable rapid problem resolution and positive experience sharing
- Multidisciplinary expertise for knowledge transfer
- Protocolized digital coaching
- One-on-one coaching with real-life examples

Improvement points

- More insight into the participants’ daily lifestyle patterns during the intervention period
- Expand the database of coaching resources (ie, materials coaches have available to support participants)

## Discussion

### Principal Results

In this study of 53 participants using the pilot version of a digitally enabled CLI-WL, we observed a mean weight loss of 3.7 kilograms (or 4.2% body weight reduction), with weight loss of more than 5 kilograms (or 5.6% body weight reduction) in individuals with obesity compared to baseline values at a median of 10 months after 16 weeks of online coaching. Both total cholesterol as well as LDL cholesterol decreased by over 10%, and systolic and diastolic blood pressure decreased by 5 and 7 mmHg, respectively. Our process evaluation analysis through surveys and interviews showed that digital implementation of a CLI-WL in the Netherlands is feasible and well-received by both participants and coaches.

### Comparison With Prior Work

Evidence for the efficacy and safety of digital (or digitally enabled) and blended CLI-WL and the reduction of cardiometabolic and cardiovascular risk has been building up over the last 5 to 10 years [43,44]. As stated previously, some of the world’s most widely adopted commercial digital therapeutic programs for weight loss have reported achieving reductions in weight varying from 2.0% to 6.8% [30,31,45]. In addition, recent reviews showed that primarily mobile digital interventions targeting overweight and obese populations with high cardiovascular risk can be at least as effective as offline programs in terms of meaningful change of lifestyle and weight loss [22]. Several offline CLI-WL programs were shown to be effective to varying degrees in the Netherlands. Across three of the four interventions available in Dutch health care, weight loss achieved at 1 year varied between 2.9 kilograms and 2.2 kilograms [22,23,46]. Only one of these interventions reported follow-up data at 2 years, where participants lost an average of only 1.5 kilograms [47]. Another study reported an average weight loss of 2.5 kilograms at 18 months [23]. While we currently do not have such long-term data, a digitally enabled intervention that was similar to this CLI-WL (with a total duration of 12 months, including a maintenance phase), delivered in a real-world context, achieved long-term weight reductions of 7.2% and 7.6% in participants with overweight and obesity, respectively [48].

While none of the currently available CLI-WLs provided data on improvement in concomitant cardiometabolic risk factors, it is worthwhile to contextualize the changes achieved in blood pressure and lipids in this pilot study. Evidence for beneficial effects of healthful lifestyle modifications on blood pressure is solid, with several studies suggesting that lifestyle adaptation is preferable to pharmacological treatment in early stages of the disease [49]. Keeping in mind the observational nature of the results reported in this study, these do surpass the results of other recently evaluated digital therapeutic tools, which yielded reductions between 2.4 and 4.3 mmHg in mean blood pressure in randomized trials [50,51]. Similarly, the effect of lifestyle programs on cholesterol levels is well-established, with reductions varying from 7% to 9% to 20% for interventions of different intensity and complexity [52]. Interestingly, for web-based interventions, meta-analyses have shown total and LDL cholesterol improvements of approximately 0.15 mmol/L [53]. When compared to the results achieved previously in another cohort of individuals who participated in an Ancora Health digital lifestyle intervention, this group of individuals with overweight and obesity showed higher baseline lipid and blood pressure values and equal or greater reductions after the program [32,33].

In light of this evidence, it is worth discussing potential reasons why this blended CLI-WL achieved better results compared to those of previous studies. In a study evaluating the features of digitally enabled weight management programs, success was linked to the ability to promote behavioral change [54]. This finding is unsurprising, but what is interesting is that the study further broke down this ability to drive behavioral change to 20 features that were essential to the program. This set of features included specific goal-setting for weight, diet, caloric balance, and physical activity, as well as educational focus on various skills such as regulating eating patterns, time management, and nutritional label reading. In addition, this program also included the development of more general skills such as learning to exercise at certain target heart rates, problem solving, stress reduction, and psychological advice on how to cope with negative thinking and social cues [54]. Interestingly, these features include some of the most positive feedback points gathered during our participant survey, such as the appreciation for the stepwise approach to behavioral change in the program, the program content being relevant and engaging, and the digital support provided by the human coaching for overcoming challenges. In the qualitative assessment, participants further mentioned highly valuing the possibility to set clear goals for the intervention and that the coaching included elements that go beyond those of regular weight loss programs, such as advice on strength training, stress management, and sleep habits.

Other studies have focused on the potential advantages of digitally enabled interventions, such as the ability for participants to access educational information more easily and at their own discretion [44,55]. Unlike offline interventions driven primarily by one health care provider with a specific focus, digitally enabled programs can easily provide information covering a range of topics required to lead a balanced lifestyle across all relevant lifestyle domains. This seems to be especially powerful when apps also provide users with tools to help them track changes in weight and BMI. For instance, in-app actions such as self-monitoring of weight and the consistency of such health behavior tracking, as well as app engagement as measured by log-ins, predicted weight loss even in older populations [44,55]. These elements again come back in the qualitative feedback from participants highlighted in Textbox 2, who highly valued the multidisciplinary nature of the content and of the coaching, and suggested that even more attention should be paid to data collected about participants’ daily lifestyle patterns. In another study comparing a mobile app–based weight loss program with a traditional offline weight loss program, success in the weight loss intervention was linked to the digitalization of components common to the traditional program [56], including providing online food diaries rather than paper diaries or when the same curriculum delivered by the dietitian in person was delivered digitally. One such app-based program featured regular contact moments with a dietitian via either text messages or video calls, while allowing them to record their food, activity, and weight, as well as providing access to educational materials and a group chat [57].

### Future Perspectives for Digital CLIs in The Netherlands

In the face of increasing rates of obesity, CVD, and (pre)diabetes, it is clear that there is a need for CLI-WLs to be deployed at scale in the Netherlands. However, current market penetration of these insurance-reimbursed programs is extremely low [58]. As the pathogenesis of obesity and (pre)diabetes is multifactorial, the etiology and environmental triggers vary among individuals; moreover, socioeconomic circumstances and barriers to lifestyle change also vary at an individual level. Yet, insurance-reimbursed CLI-WL programs currently offered in the Dutch market lack a data-driven approach to translating user profiles to personalized care pathways, with coaches bearing the burden of translating one-size-fits-all guidelines to a user’s context through their consultations. As suggested by the health care professionals interviewed in this study, supporting the delivery of CLI-WL programs with eHealth solutions such as those described in this research could help tailor the intervention to individual characteristics and needs, as well as tackle the practical issues of lack of time and capacity faced by care providers.

In fact, the Dutch National Institute for Public Health and the Environment (Rijksinstituut voor Volksgezondheid en Milieu [RIVM]) defines several important, evidence-based factors for the successful deployment of a (combined) lifestyle intervention program [59]. Interestingly, although these recommendations have been designed for offline programs, some of them are much more easily achievable with digitally enabled interventions. For instance, the RIVM recommends that participants of a CLI-WL who fail to achieve significant weight loss in the first month should receive increased support, since early weight loss is an important predictor of long-term success. Through its digital capabilities, the CLI-WL described herein allows for weekly monitoring of progress and enables coaches to review data and progress remotely. In addition, coaches are also able to contact the participant proactively to understand and address barriers in the absence of progress. Importantly, one full-time coach can effectively coach more than 100 participants across multiple regions while upholding the recommendation of 15 to 20 minutes of coaching time weekly. This allows for greater reach than possible with offline lifestyle interventions, which are often episodic, delivered at low frequency, and limited to participants within close proximity to the health coach. Importantly, a recent Dutch study showed that the Dutch population is generally sufficiently technology-literate, even across socioeconomic strata, and welcoming of digital technologies, which encourages the deployment of these interventions [60]. Lastly, the RIVM underscores the need for participants to develop self-efficacy skills to sustain behavioral change over the long term. This is in line with previous studies demonstrating that interventions applying theoretical frameworks or models for behavioral change—some of which were technology-based—were more effective at increasing adherence to healthy lifestyle habits than standard advice [61,62]. This program represents the first instance in which the FBM was used as part of a CLI-WL. The positive health outcomes and subjective feedback from users and health care professionals suggest that this approach holds promise and ought to be further explored for future iterations.

### Strengths and Limitations

This study has several limitations. The first is the observational nature of the study. The second limitation is the small sample size of the remeasured population. Third, participants were not followed up for the duration of a standard CLI-WL (2 years); rather, these preliminary outcomes were reported after a median of 10 months after the 16-week intervention. As such, the results of the 16-week program should be compared with caution to those of other CLI-WLs that maintained participants engaged for the entirety of the 2-year period. Fourth, participants who could not return to the health center for a remeasurement were asked to self-report their weight after 10 months. As for all self-reported outcome measures, and despite careful instructions provided by the coach, measurement errors cannot be excluded. We are currently evaluating several possibilities to overcome this, such as the delivery of a personalized program box with a connected scale and wearable device. Lastly, the goal of CLI-WLs is to provide sustained positive health outcomes after 2 years (which is also the duration of the insured intervention in public health programs). Therefore, our 10-month results show that the weight loss can be sustained for a medium-long period of time, but we do not yet have data of 2-year follow-up for these participants. More research will also be conducted to evaluate the impact of interventional elements on engagement and health outcomes, such as to identify which elements are most effective for a digitally enabled CLI-WL.

Conversely, one strength of the study is that medication information was gathered at baseline and follow-up. This allows us to verify that weight loss and the improvements reported in lipids and blood pressure did not come from cholesterol- or blood pressure–lowering medications. In addition, by gathering detailed feedback from a majority of participants, we were able to identify critical points for improvement, which contributed to the further maturing of the intervention and informed the development of the mobile app designed to support it. Lastly, this is the first study to report on a real-world application of a digitally enabled intervention targeting health behavior change to promote weight loss in a cohort of Dutch adults with overweight and cardiometabolic risk factors.

### Conclusions

In conclusion, a digitally enabled CLI showed sustained weight loss in individuals with overweight and obesity at the 10-month follow-up. Albeit preliminary, these findings suggest that a behavioral lifestyle program with a digital component deployed for this intervention can achieve greater weight loss than previously reported for currently available offline CLI-WLs in the Netherlands. Whereas larger-scale observational studies and randomized trials of other digital interventions have been conducted and shown evidence of effectiveness for digitally enabled CLI-WL in other countries, this study is a demonstration that such an approach can also be effective in the Netherlands. This is an important first step, as the feasibility of these programs remains a hot topic in public health discussions. Based on the qualitative feedback collected from pilot participants and health care professionals, this first version of the program is being further developed and evaluated. Evidence from future studies in a Dutch population may help elucidate the mechanisms behind the effectiveness of a digitally enabled CLI-WL.

## Abbreviations

CLI: combined lifestyle intervention
CLI-WL: combined lifestyle intervention for weight loss
CVD: cardiovascular disease
FBM: Fogg Behavioral Model
LDL: low-density lipoprotein
RIVM: Rijksinstituut voor Volksgezondheid en Milieu (Dutch National Institute for Public Health and the Environment)

## Data Availability

The data sets generated during and/or analyzed during this study are available from the corresponding author on reasonable request.
